# Hepatitis E Virus Genotype 4 Sequences Detected in Sewage from Treatment Plants of China

**DOI:** 10.1007/s12560-016-9276-y

**Published:** 2017-01-21

**Authors:** Heng Li, Wei Li, Ruiping She, Liang Yu, Qiaoxing Wu, Jingling Yang, Fengjiao Hu, Majid Hussain Soomro, Ruihan Shi, Wenzhuo Hao, Yue Zhao, Jingjing Mao

**Affiliations:** 1 Laboratory of Veterinary Pathology and Public Health, College of Veterinary Medicine, China Agricultural University, Yuanmingyuan WestRoad 2#, Haidian District, Beijing, 100193 China; 2 Shenzhen Urban Wastes Disposal & Recycling Center, Yuyuan Road 1#, Longgang District, Shenzhen, 518000 China; 3 Laboratory of Animal Pathology & Public Health, Key Laboratory of Zoonosis of the Ministry of Agriculture College of Veterinary Medicine, China Agricultural University, Beijing, 100193 China

**Keywords:** Hepatitis E virus, Genotype 4, Urban sewage

## Abstract

The aim of this study was to investigate the occurrence of hepatitis E virus (HEV) in sewage samples in Shen Zhen, China. Sewage samples were collected from 152 sewage plants including livestock sewage, domestic sewage and treated sewage from May to July of 2015. Two of 152 samples were HEV positive (1.32%) from the livestock sewage plants. Partial ORF2 fragments of HEV were sequenced and a phylogenetic tree was constructed using MEGA5.1. Blast and phylogenetic analyses showed that both of these two sequences belonged to HEV Genotype 4. To the best of our knowledge, this is the first study on the molecular characterization of HEV in wastewater in China and the first time to detect Genotype 4 in the sewage. Results from this study indicate that the possibilities of sporadic infections of HEV should be emphasized because virus still has the possibility to be circulating in the sewage in China.

Hepatitis E (HE) is an acute self-limiting disease caused by a non-enveloped, positive-sense, single-stranded RNA virus designated ‘‘hepatitis E virus’’ (HEV) (Mao et al. 2013). Polluted food and water can be the sources of infection and waterborne transmission is the chief type for HEV spreading (Yugo and Meng 2013). Recently, divergent HEV strains have been discovered in the sewages in different countries from all over the world. In the present study, we aimed to provide preliminary information regarding the occurrence and molecular characterization of HEV in different sewage samples in Shen Zhen (China), through the monitoring of 152 sewage samples collected including livestock sewage, domestic sewage and treated sewage.

The samples, 51 for livestock sewage, 51 for domestic sewage, and 50 for treated sewage were collected every month from three sewage treatment plants between May and July 2015. One hundred and twenty farms were included in the study.

The sewage samples (2 l in total) were collected and divided into aliquots of 10 ml, then 10% (w/v) PEG 6000 was added to each sample along with sodium chloride to the final concentration of 0.4 M. After centrifuging, concentrated sewage samples (1 ml each) were extracted and stored at −80 °C until they were tested. Total RNA was extracted from sewage samples using the UltraPure™ RNA Kit (CWBIO,Beijing, China), according to the manufacturer’s instructions. Then the extracted RNA was used for complementary DNA (cDNA) synthesis using the HiFiScript 1st Strand cDNA Synthesis Kit (CWBIO, Beijing, China) at 42 °C for 50 min and 70 °C for 15 min. Nested PCR was carried out to amplify a partial fragment of ORF2 (nt 5,983-6,349) of the HEV genome. The primers sets P1 [5′-AATTATGCYCAGTAYCGRGTTG-3′] and P2 [5′-CCCTTRTCYTGCTGMGCATTCTC-3′] were used for the first round, P3 [5′-GTWATGCTYTGCATWCATGGCT-3′] and P4 [5′-AGCCGACGAAATCAATTCTGTC-3′] for the second round (Tam et al. 1991). The amplification parameters for both rounds of PCR were the same: 95 °C for 7 min, followed by 35 cycles of 94°Cfor 1 min, 42 °C for 1 min, and 72 °C for 2 min, with a final extension step at 72 °C for 10 min. Fragment sizes were compared ranging from 100 to 2000 bp and The HEV amplicon was 348 bp.

The PCR results indicated that two (1.32%) of the 152 samples from the sewage plant were positive for HEV. In order to further identify the genetic relationship between this virus and other known HEV strains, we evaluated the relatedness of sequences deposited in public databases by BLAST. Blast analysis exhibited that it showed 95 and 99% identities to the sequences detected from the swine in Guangdong province, China. Also by using the software of MEGA5.1, a neighbor-joining tree was constructed with bootstrap values calculated from 1000 replicates. According to the neighbor-joining tree, these two kinds of HEV detected from the sewage samples were both Genotype 4 (GenBank accession nos. KT008933), and were closely related to the AJ272108 and AY594199 (Fig. 1).

**Fig. 1:**
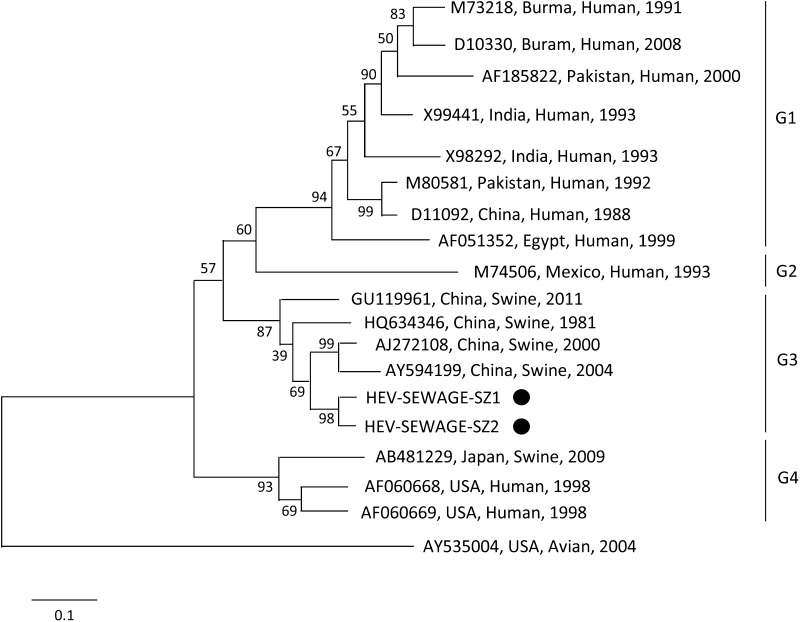
Phylogenetic analysis based on ORF2 (348 nt) depicting the genetic relationship between our strain from this study. A neighbor-joining tree was constructed with bootstrap values calculated from 1000 replicates. Isolates used for comparative analysis were Burma (GenBank Accession No. M73218, D10330), Pakistan (AF185822), India (X99441), India (X98292), Pakistan (M80581), China (D11092), Eygypt (AF051352), Mexico (M74506), China (GU119961), China (HQ634346), China (AJ272108), China (AY594199), Japan (AB481229), USA(AF060669), USA (AF060668), and Avian HEV (AY535004)

Since the first swine strain was characterized from a pig in the United States, HEV infection in swine appears to be an important cause of acute viral hepatitis worldwide (Meng et al. 1997). Nowadays, China is commonly considered as an endemic area for Hepatitis E (Xiao et al. 2012). It is well known that Genotypes 3 and 4 are zoonotic and are responsible for sporadic and autochthonous infections in both humans and swine (Mao et al. 2013). Meanwhile, swine have been known to serve as a reservoir species for HEV transmission to humans (Yugo and Meng 2013). In our research, we collected the sewage from the sewage treatment plants of the city and detected HEV successfully. However, there is still lack of concrete data on the concentrations of the detected sequences. Since China is a large country for pig industry and pork production, testing HEV concentrations in sewage seems to be particularly important. Next step, we would like to do more research on the concentrations of HEV in the sewage.

According to the reports, raw sewage water has been shown to contain HEV strain related to the circulating in humans and animals closely in both developed countries and the developing countries (Yugo and Meng 2013). In Netherlands (Rutjes et al. 2009) and Japan (Ishida et al. 2012), HEV of Genotype 3 had been detected in the sewage samples. Meanwhile, a HEV of Genotype 1 was detected from the sewage samples in Italy between 2008 and 2009 (Iaconelli et al. 2015). Moreover, the research in Tunisia also showed the positive results in HEV of Genotype 1 and Genotype 3 in the sewage, even though pork products are not consumed in this country (Beji-Hamza et al. 2015). On the other hand, a considerable amount of experimental studies have been devoted to elucidate the possibility for the transmission of HEV from swine to human, which indicating the public sanitation significance of zoonosis for human beings again. For example, sporadic human cases of acute HEV infection linked with consumption of raw or insufficiently cooked meat have been reported in Japan (Tei et al. 2004). In France, the isolation of the viruses have the similar sequences between a patient and his pet pig, which also suggests the possible route of transmission was from pig to human (Christophe et al. 2007). Combined with previous study, we assume that in our study fecal specimens from the HEV-infected swine in the piggeries of suburbs would be the primary factor to pollute the untreated sewage effluents of the city. Then human could be infected with HEV by consuming the animal reservoir. Although direct mechanism of HEV transmission from animals to humans is lacking, swine recognized as a major reservoir for HEV transmission to humans has gradually become an undoubted fact (Fig. 2) (Williams et al. 2001, Fauci et al. 2010, Xiao et al. 2012).

**Fig. 2:**
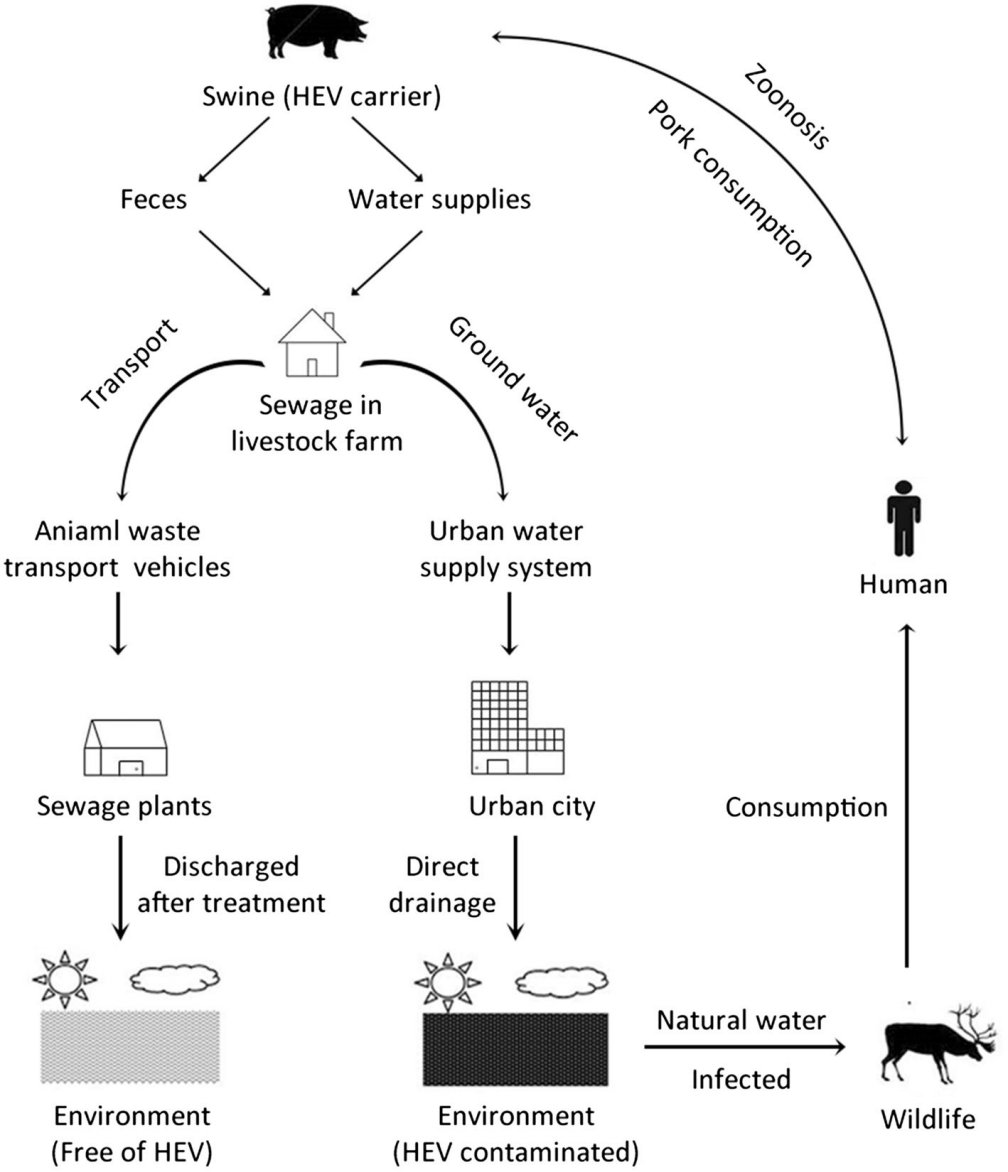
HEV-infected swine in the livestock farm would be the primary factor to contaminated the untreated sewage effluents of the city and then HEV will be circulating in the environment

To the best of our knowledge, this is the first study on the molecular characterization of HEV in wastewater in China and the first time to detected Genotype 4 in the sewage. More studies are necessary to gather information on the occurrence and diversity of the strains circulating in humans and animals in order to gain a better understanding of the epidemiology of HEV and assess the public health risk linked to contamination of aquatic environments. Control strategies and tactics should be published for the prevention and treatment of HEV thoughtfully.

## Abbreviations

HEV: Hepatitis E virus
HE: Hepatitis E
RT-nPCR: Reverse transcription nest PCR
ORF2: Open reading frame 2
